# Use of Coronary Computed Tomographic Angiography to Guide Management of Patients With Coronary Disease

**DOI:** 10.1016/j.jacc.2016.02.026

**Published:** 2016-04-19

**Authors:** Michelle C. Williams, Amanda Hunter, Anoop S.V. Shah, Valentina Assi, Stephanie Lewis, Joel Smith, Colin Berry, Nicholas A. Boon, Elizabeth Clark, Marcus Flather, John Forbes, Scott McLean, Giles Roditi, Edwin J.R. van Beek, Adam D. Timmis, David E. Newby, SCOT-HEART Investigators

**Affiliations:** a British Heart Foundation Centre for Cardiovascular Science, University of Edinburgh, Edinburgh, United Kingdom; b Centre for Population Health Sciences, University of Edinburgh, Edinburgh, United Kingdom; c Health Economics Research Centre, University of Oxford, Oxford, United Kingdom; d Institute for Cardiovascular and Medical Sciences, University of Glasgow, Glasgow, United Kingdom; e Norwich Medical School, University of East Anglia, Norwich, United Kingdom; f Health Research Institute, University of Limerick, Limerick, Ireland; g National Health Service, Fife, United Kingdom; h William Harvey Research Institute, Queen Mary University of London, London, United Kingdom

**Keywords:** angina pectoris, invasive coronary angiography, myocardial infarction, preventive therapy, CCTA, coronary computed tomography angiography, HR, hazard ratio, IQR, interquartile range, OR, odds ratio

## Abstract

**Background:**

In a prospective, multicenter, randomized controlled trial, 4,146 patients were randomized to receive standard care or standard care plus coronary computed tomography angiography (CCTA).

**Objectives:**

The purpose of this study was to explore the consequences of CCTA-assisted diagnosis on invasive coronary angiography, preventive treatments, and clinical outcomes.

**Methods:**

In post hoc analyses, we assessed changes in invasive coronary angiography, preventive treatments, and clinical outcomes using national electronic health records.

**Results:**

Despite similar overall rates (409 vs. 401; p = 0.451), invasive angiography was less likely to demonstrate normal coronary arteries (20 vs. 56; hazard ratios [HRs]: 0.39 [95% confidence interval (CI): 0.23 to 0.68]; p < 0.001) but more likely to show obstructive coronary artery disease (283 vs. 230; HR: 1.29 [95% CI: 1.08 to 1.55]; p = 0.005) in those allocated to CCTA. More preventive therapies (283 vs. 74; HR: 4.03 [95% CI: 3.12 to 5.20]; p < 0.001) were initiated after CCTA, with each drug commencing at a median of 48 to 52 days after clinic attendance. From the median time for preventive therapy initiation (50 days), fatal and nonfatal myocardial infarction was halved in patients allocated to CCTA compared with those assigned to standard care (17 vs. 34; HR: 0.50 [95% CI: 0.28 to 0.88]; p = 0.020). Cumulative 6-month costs were slightly higher with CCTA: difference $462 (95% CI: $303 to $621).

**Conclusions:**

In patients with suspected angina due to coronary heart disease, CCTA leads to more appropriate use of invasive angiography and alterations in preventive therapies that were associated with a halving of fatal and non-fatal myocardial infarction. (Scottish COmputed Tomography of the HEART Trial [SCOT-HEART]; NCT01149590)

Patients who present with chest pain of suspected cardiac origin require accurate and timely diagnosis to guide the implementation of appropriate investigations and therapeutic interventions. Current U.S. (1) and European (2) guidelines describe a range of potential noninvasive imaging modalities to investigate patients with suspected stable angina pectoris due to coronary heart disease. However, there is little definitive or consistent evidence of superiority of 1 imaging modality over another, and none has yet demonstrated improvements in downstream clinical outcomes attributable to better diagnostic performance. Moreover, American guidelines (1) specifically favor stress testing as the initial diagnostic test of choice and reserve coronary computed tomography angiography (CCTA) for patients who are unable to undergo stress testing.

The SCOT-HEART (Scottish COmputed Tomography of the HEART) trial showed that, when used in addition to standard care, CCTA markedly clarified the diagnosis for patients with suspected angina due to coronary heart disease (3). This diagnostic improvement was associated with alterations in downstream investigations and treatments and with potential improvements in clinical outcome. However, whether CCTA-guided changes in diagnosis led to appropriate improvements in invasive coronary angiography and initiation of preventive treatments, and whether these changes could be attributable to an improvement in clinical outcome, has not been explored.

It would be neither practical nor ethical to undertake invasive coronary angiography in all patients within a large trial of a noninvasive diagnostic test for angina pectoris due to coronary heart disease. However, a reasonable proxy for the assessment of diagnostic accuracy is to compare the rates of normal coronary arteries or obstructive coronary artery disease at the time of invasive coronary angiography. To assess the appropriateness of therapy would again be inferential and requires the assessment of improvements in clinical outcomes directly attributable to coronary heart disease. For these clinical improvements to occur, the changes in management consequent on the diagnostic test have to be implemented and temporally associated with any observed benefits. Clearly, it is not sufficient for the test to be merely performed.

In this study, we aimed to assess the diagnostic utility of CCTA against the findings at invasive coronary angiography, and to investigate the timing and therapeutic implementation of CCTA-guided changes in preventive treatment. Finally, we explored the beneficial effects of these investigative and therapeutic implementations on coronary heart disease events.

## Methods

### Study design

The SCOT-HEART study was a prospective, open-label, parallel group, multicenter, randomized controlled trial that assessed the role of CCTA in patients with suspected angina due to coronary heart disease who attended a cardiology clinic. The study design has previously been described in detail (4) and the primary study findings published (3). The study was conducted in accordance with the Declaration of Helsinki and with research ethics committee approval.

### Participants

Participants were recruited from dedicated cardiology chest pain clinics where they were referred with suspected angina due to coronary heart disease. A total of 4,146 patients age 18 to 75 years were recruited as described previously (4). Participants were randomized 1:1 to standard care or standard care plus ≥64-slice CCTA using a web-based randomization system with minimization for age, sex, body mass index, diabetes, history of coronary heart disease, atrial fibrillation, and the baseline diagnosis of angina due to coronary heart disease. Standard of care included stress testing according to established local clinical protocols.

### Computed tomography coronary angiography

Participants in the CCTA arm of the study underwent coronary artery calcium scoring and CCTA using 64-detector (Brilliance 64, Philips Healthcare, North Andover, Massachusetts; or Biograph mCT, Siemens, Erlangen, Germany) or 320-detector (Aquilion One, Toshiba Medical Systems, Tochigi, Japan) row scanners at 1 of 3 imaging sites. Computed tomography images were assessed by ≥2 trained observers (4) with excellent reproducibility (5). The overall results of the scan were defined as normal (<10% luminal cross-sectional area), nonobstructive (mild 10% to 49%, moderate 50% to 70%), or obstructive (>70%) coronary artery disease. Overall, obstructive coronary artery disease was defined as a cross-sectional luminal stenosis of >70% in ≥1 major epicardial vessel or >50% in the left main stem.

### Invasive coronary angiography and coronary revascularization

The Scottish national electronic health record system was used to obtain information on the timing and results of subsequent invasive coronary angiography and coronary revascularization (percutaneous coronary intervention or coronary artery bypass graft surgery). All procedures were performed according to routine clinical practice by the attending clinicians, interventional cardiologists, and surgeons. The results of invasive coronary angiograms were categorized as normal, nonobstructive coronary artery disease, or obstructive coronary artery disease. Obstructive coronary artery disease was classified as a lesion with >50% diameter stenosis and/or abnormal fractional flow reserve. The time interval between the initial clinic attendance and invasive coronary angiography or coronary revascularization procedure was recorded.

### Medications

The Scottish national community drug-prescribing database of the Information Services Division of the National Health Service (NHS) Scotland maintains a detailed record of all NHS prescriptions dispensed in the community, which are linked to individual patient identifiers. All of these prescriptions are dispensed by community pharmacies, dispensing doctors, and a small number of specialist appliance suppliers. Preventive medications were categorized into antiplatelet, statin, and angiotensin-converting enzyme inhibition therapies. We extracted information on which medications were obtained and the timing of dispensing from the pharmacist. The time interval between randomization to the dispensing of the prescription was determined.

### Outcomes

The Scottish national morbidity record with linkage to the General Registers Office was used to obtain information on the long-term outcomes of the study including death, myocardial infarction (MI), and cerebrovascular disease. This information also was obtained from the Information Services Division of NHS Scotland and, where appropriate, confirmed with review of the electronic patient health records. The inpatient and day-case national dataset collects episode-level data across all hospitals in Scotland. This includes discharge diagnostic codes using the International Classification of Disease, 10th Revision, system and operational codes using the Office of Population Censuses and Surveys’ Classification of Interventions and Procedures, as described previously (3). Categorization of cardiovascular events and cause of death was adjudicated blinded to trial allocation.

### Statistical analysis

Statistical analysis was performed using SAS version 9.4 (SAS Institute, Cary, North Carolina). Data were analyzed using Cox proportional hazards regression and presented graphically using Kaplan-Meier plots. Logistic regression models were used to compare the changes in prescriptions between the 2 treatment arms. All analyses were adjusted for center and minimization variables excluding the baseline diagnosis. To examine whether there was a change in the HR after 50 days, a randomized treatment by time interaction term was added in to the model of fatal and nonfatal MI. Cost analyses were performed as detailed in the Online Appendix. Analyses were post-hoc, and statistical significance was defined as a 2-sided p < 0.05.

## Results

Of the 4,146 patients recruited into the study, 2,073 were assigned to standard care and 2,073 to standard care plus CCTA. Participants were age 57 ± 10 years and had a body mass index of 30 ± 6 kg/m^2^; 56% (n = 2,325) were male. Patient characteristics have previously been described in detail (3) and patients were followed for a median of 20 months (range 3 to 49 months).

### Coronary computed tomography angiography

**Figure 1:**
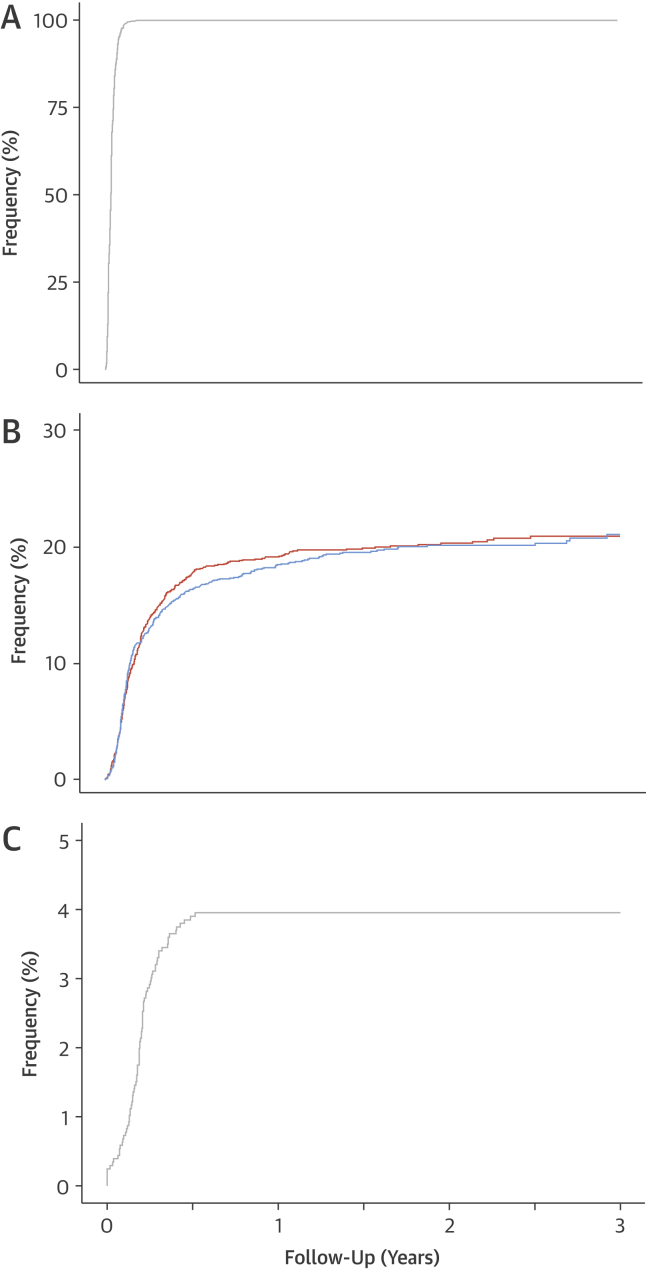
Timing of Noninvasive and Invasive Coronary Angiography Cumulative events from clinic consultation to the conduct of: **(A)** coronary computed tomography angiography (CCTA) in patients receiving the trial intervention, **(B)** invasive coronary angiography by trial allocation, and **(C)** new invasive coronary angiography consequent on the CCTA findings. Hazard ratio: 1.06 (95% confidence interval: 0.92 to 1.22); p = 0.451 for rates of invasive coronary angiography between CCTA + standard care and standard care alone. **Orange line** indicates CCTA + standard care. **Blue line** indicates standard care alone.

Of those 2,073 patients assigned to standard care plus CCTA, 1,778 (86%) underwent CCTA with the remainder (n = 295; 14%) defaulting or not completing the scan. The median interval between randomization and CCTA was 12 days (interquartile range [IQR]: 7 to 18 days) (Figure 1). CCTA demonstrated normal coronary arteries in 654 (37%), mild nonobstructive (10% to 50% luminal stenosis) disease in 372 (21%), intermediate nonobstructive (50% to 70%) disease in 300 (17%), and obstructive (>70%) disease in 452 (25%) participants. By 6 weeks, CCTA was associated with a higher rate of cancellation (29 vs. 1; odds ratio [OR]: 30.94 [95% confidence interval (CI): 4.20 to 227.72]; p = 0.0008) and the request of new (94 vs. 8; OR: 12.85 [95% CI: 6.21 to 26.59]; p < 0.0001) invasive coronary angiograms that were performed 74 days (IQR: 53 to 95 days) after clinic consultation (Figure 1).

### Invasive coronary angiography

Over the total trial follow-up period, there were no differences in the number of invasive angiographic procedures (409 vs. 401; HR: 1.06 [95% CI: 0.92 to 1.22]; p = 0.451) (Figure 1). However, invasive coronary angiography was less likely to demonstrate normal coronary arteries (20 vs. 56; HR: 0.39 [95% CI: 0.23 to 0.68]; p < 0.001) and more likely to show obstructive coronary artery disease (283 vs. 230; HR: 1.29 [95% CI: 1.08 to 1.55]; p = 0.005) in patients assigned to CCTA. There was no difference in the rates of nonobstructive coronary artery disease (97 vs. 107; HR: 1.01 [95% CI: 0.76 to 1.35]; p = 0.926).

Overall, there was an apparent trend for more coronary revascularization procedures (233 vs. 201; HR: 1.20 [95% CI: 0.99 to 1.45]; p = 0.061) to occur in those allocated to CCTA. Coronary revascularization (percutaneous coronary intervention or coronary artery bypass grafting) was performed 76 days (IQR: 56 to 118 days) after clinic attendance.

### Preventive medications

**Figure 2:**
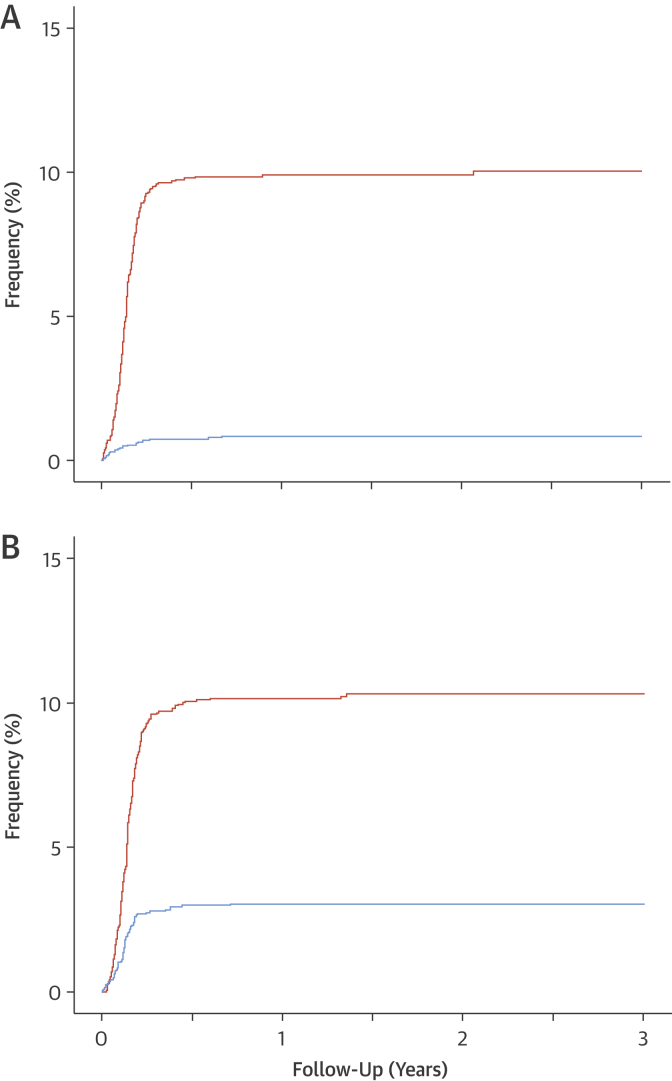
Timing of Initiation of New Preventive Therapies Cumulative events from clinic consultation to the dispensing of: **(A)** antiplatelet therapy (hazard ratio: 12.17 [95% confidence interval: 7.52 to 19.71]; p < 0.0001) and **(B)** statin therapy (hazard ratio: 3.49 [95% confidence interval: 2.63 to 4.42]; p < 0.0001), according to trial allocation. **Orange line** indicates coronary computed tomography angiography + standard care. **Blue line** indicates standard care alone.

Compared with standard care, clinicians were more likely to recommend cancellation (77 vs. 8; OR: 10.75 [95% CI: 5.51 to 22.42]; p < 0.001) and initiation (293 vs. 84; OR: 4.12 [95% CI: 3.19 to 5.33]; p < 0·001) of preventive therapies after CCTA. This was attributable to the changes in the diagnosis consequent on the CCTA result (Online Table 1). New preventive therapies were composed of antiplatelet (n = 220 vs. 33; HR: 12.17 [95% CI: 7.52 to 19.71]; p < 0.001), statin (n = 226 vs. 80; HR: 3.49 [95% CI: 2·63 to 4.62]; p < 0.001), and angiotensin-converting enzyme inhibition (n = 19 vs. 1; HR: 10.73 [95% CI: 1·38 to 83.25]; p = 0.0232) therapies. After clinic attendance, these recommendations were generally implemented (283 vs. 74; HR: 4.05 [95% CI: 3.13 to 5.23]; p < 0.001) and associated with the issuing of 347 new prescriptions at a median of 50 days (IQR: 36 to 66 days) for antiplatelet, 52 days (IQR: 37 to 66 days) for statins, and 48 days (IQR: 31 to 89 days) for angiotensin-converting enzyme inhibition therapies (Figure 2).

### Clinical outcomes

**Figure 3:**
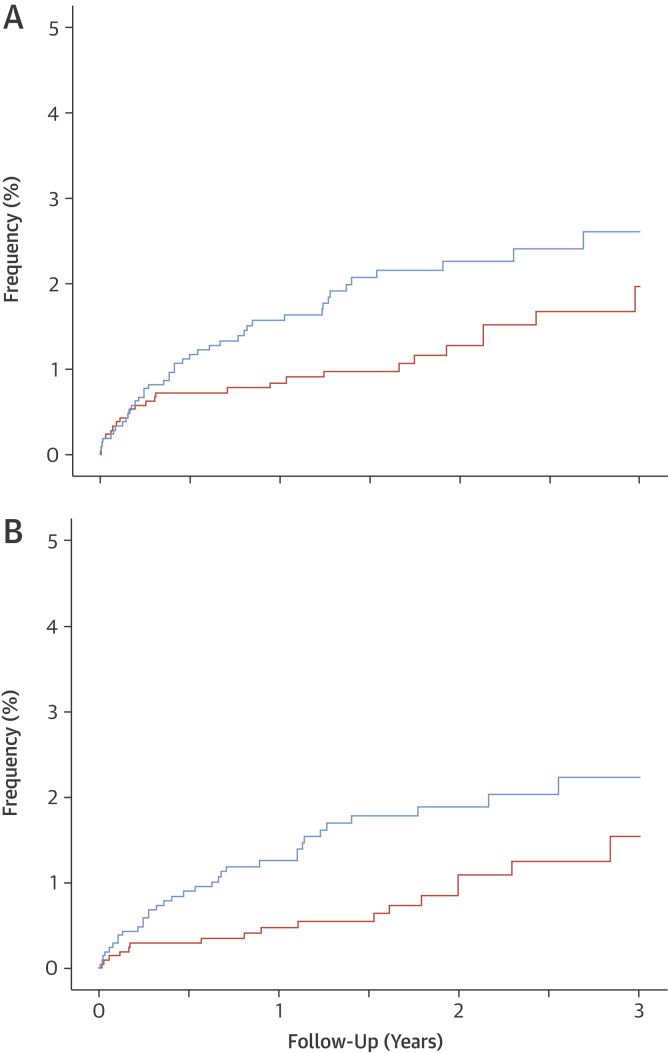
Fatal and Nonfatal Myocardial Infarction With and Without the 50-Day Implementation Delay Cumulative fatal and nonfatal myocardial infarction over 3 years of follow-up **(A)** including (26 vs. 42; hazard ratio: 0.62 [95% confidence interval: 0.38 to 1.01]; p = 0.0527) and **(B)** excluding (17 vs. 34; hazard ratio: 0.50 [95% confidence interval: 0.28 to 0.88]; p = 0.0202) the first 50 days after clinic consultation. **Orange line** indicates coronary computed tomography angiography + standard care. **Blue line** indicates standard care alone.

As anticipated, rates of MI were highest in those with obstructive coronary heart disease and were intermediate in those with nonobstructive disease (Online Figure 1). Compared with standard care, the rates of fatal and nonfatal MI appeared to be reduced in patients undergoing CCTA (26 vs. 42; HR: 0.62 [95% CI: 0.38 to 1.01]; p = 0.0527). Although there was no statistically significant interaction between treatment and time (p = 0.324), and the results are on the basis of a small number of events, there was an apparent separation of the event curves several weeks after randomization (Figure 3A). Given that changes in clinical events cannot be attributable to the CCTA alone, we explored the timing of treatment changes consequent on the CCTA using the dispensing of preventive therapies. Prior to the median time for the implementation of preventive therapies (0 to 49 days), there were no differences in the rates of fatal and nonfatal MI according to randomized trial allocation (9 vs. 8; HR: 1.09 [95% CI: 0.42 to 2.83]; p = 0.8589). Analysis of events at ≥50 days suggested a halving in the rate of fatal and nonfatal MI (17 vs. 34; HR: 0·50 [95% CI: 0.28 to 0.88]; p = 0.0202) (Figure 3, Online Table 2), and nonfatal stroke, and nonfatal and fatal MI (22 vs. 40; HR: 0.55 [95% CI: 0.33 to 0.91]; p = 0.0245) in patients allocated to CCTA compared with those assigned to standard care.

### Health care costs

The cumulative mean cost of treatment over 6 months was higher in the CCTA group than for standard care alone: $1,900 versus $1,438, difference $462 (95% CI: $303 to $621) (Table 1). The total cost difference is attributable to the direct costs of CCTA itself and was robust for a range of assumptions that varied with the mean unit cost of CCTA. There were no differences in the costs attributed to outpatient, day-case, or inpatient services, reflecting the broadly similar rates of investigations and procedures following randomization to either CCTA or standard care alone. Overall, there were no differences in the downstream costs: $89 (95% CI: −$69 to $248) (p = 0.269).

**Table 1:** Cumulative 6-Month Resource Use and Costs

	Coronary Computed Tomography Angiography (n = 2,073)	Standard Care (n = 2,073)	Difference (95% Confidence Intervals)	p Value∗
Investigation				
Invasive coronary angiography	17.5	16.3	1.2 (−1.0 to 3.5)	0.28
Coronary revascularization				
Percutaneous coronary intervention	8.9	7.1	1.8 (−0.4 to 4.0)	0.11
Coronary artery bypass graft surgery	1.9	1.8	0.1 (−0.1 to 0.0)	0.73
Total hospital length of stay, days	0.3 ± 0.8	0.3 ± 0.8	0.0 (−0.1 to 0.0)	0.86
Component costs, USD				
Coronary computed tomography angiography	372 ± 163 [416]	0 ± 16 [0]	372 (363 to 378)	<0.001
Outpatient services	219 ± 655 [0]	192 ± 584 [0]	27 (−10 to 66)	0.16
Day-case services	890 ± 1,196 [0]	827 ± 1,189 [0]	63 (−10 to 136)	0.09
Inpatient services	379 ± 1,906 [0]	379 ± 1,864 [0]	1 (−113 to 116)	0.98
Medications	52 ± 67 [0]	50 ± 70 [0]	1 (−3 to 6)	0.50
Total 6-month costs, USD	$1,900 ± $2,642 [$552]	$1,438 ± $2,581 [$86]	$462 ($303 to $621)	<0.001

Values are % or mean ± SD [median], unless otherwise specified.

∗ Student *t* test.

## Discussion

We have previously reported that CCTA clarifies the diagnosis, changes treatments and investigations, and may improve outcomes in patients with suspected angina pectoris due to coronary heart disease. However, whether these changes in management were appropriate and could be plausibly related to apparent improvements in outcomes remained to be established. Here, we have demonstrated that the changes in the selection of patients for invasive coronary angiography were appropriate, resulted in markedly lower rates of normal coronary angiography and a higher rate of obstructive coronary artery disease, and apparently led to more coronary revascularization procedures. In addition, the timing of the subsequent changes in preventive therapies coincided with the apparent divergence of event rates for fatal and nonfatal MI. We conclude that the changes in diagnosis consequent on CCTA led to appropriate changes in the selection of patients for invasive coronary angiography and the more effective implementation of preventive therapies, which were associated with a halving in the subsequent rate of fatal and nonfatal MI.

Studies addressing the diagnostic accuracy of CCTA are well described 6, 7, and the major strength of CCTA relates to exclusion of coronary heart disease. Here, we have demonstrated that the addition of CCTA to routine clinical assessment of patients with suspected angina pectoris secondary to coronary heart disease leads to a nearly 3-fold reduction in the rates of normal invasive coronary angiography. These reductions in the rates of normal coronary angiography were consistent with those observed in the PROMISE (PROspective Multicenter Imaging Study for Evaluation of chest pain) trial (8), although the proportionate reductions were even greater in the present study. Importantly, we also found that not only did the rate of normal coronary arteries fall, but also the rate of obstructive coronary artery disease increased in those undergoing invasive coronary angiography. This suggests better use of invasive angiography, especially as the rate of coronary revascularization was high in those for whom CCTA had changed the initial diagnosis and suggested the presence of obstructive disease. Given the potentially greater hazards and costs of invasive angiography, our findings indicate that CCTA is as an effective and readily applicable gatekeeper for the conduct of invasive coronary angiography with a view to coronary revascularization in patients with suspected angina pectoris due to coronary heart disease.

Models have suggested that CCTA can be used to select asymptomatic individuals at higher risk of cardiovascular events for preventive therapy 9, 10. Indeed, a recent substudy of the CONFIRM (COronary computed tomography angiography evaluatioN For clinical outcomes InteRnational Multicenter) registry suggested that the baseline use of statin therapy was associated with lower mortality in asymptomatic patients with nonobstructive or obstructive coronary artery disease identified by CCTA, whereas mortality was unaffected in those with normal coronary arteries (11). In contrast, McEvoy et al. (12) reported that although aspirin and statin therapy use was increased in 1,000 asymptomatic patients who underwent CCTA for the identification of coronary artery disease, mortality was no different than in a referent control group that had not undergone CCTA. For symptomatic patients with suspected coronary artery disease, the presence and severity of coronary artery disease identified on CCTA is associated with the increased use of preventive therapies such as aspirin and statin therapies 13, 14 as well as lifestyle modification 14, 15. A single-center registry of 8,372 patients with nonobstructive coronary artery disease identified by CCTA also showed that statin therapy was associated with lower mortality, but aspirin therapy was only associated with lower mortality in high-risk patients 16, 17. However, all of these observational studies have many potential biases, including case selection bias and confounding by treatment allocation. Our study avoids these biases through the conduct of a randomized controlled trial of all patients attending the cardiology clinic for suspected angina pectoris due to coronary heart disease. Because the allocation of imaging was randomized, the subsequent downstream alterations in treatment can be attributable to the imaging intervention. In addition, although the SCOT-HEART trial event rates were relatively modest, the rates of fatal and nonfatal MI in trial participants were greater than those observed in asymptomatic individuals 9, 16 and similar to those in symptomatic patients with stable disease 18, 19. Finally, the benefits of preventive therapies are greatest in treatment naïve patients with new-onset angina pectoris, especially given that the latter is associated with a potentially more unstable course and represents an intermediate-severity risk group 1, 20.

We collected data from the attending clinicians 6 weeks after the clinic consultation to ascertain the recommended changes in management and treatment of patients in both allocated groups. Compared with standard care, there were marked differences in the recommendations of how the patients were to be managed and treated following CCTA (3). However, were these changes implemented, and if so, when? We explored both the implementation and the timing of these changes using national electronic health records and prescribing data. This was a major strength of our study and has not previously been employed in other trials of this type. We were able to determine the exact date of the dispensing of medications to individual patients consequent on the attending clinicians recommendations. We described the inevitable delay consequent on the time taken to undertake the CCTA, the report to be issued, the attending clinician to review and act on the report, the primary care physician to implement the recommendation, and ultimately, for a prescription to be submitted and medication dispensed to the patient. The median time delay from clinic consultation to the dispensing of medications was approximately 50 days for aspirin, statin, and angiotensin-converting enzyme inhibitor therapies.

There are currently no noninvasive imaging strategies that have been shown to reduce cardiovascular events in a randomized controlled trial. This is perhaps not surprising, because there are multiple steps from diagnosis to treatment to clinical events, and it is challenging to demonstrate the effectiveness of an imaging strategy on hard clinical outcomes (21). However, we have reported that the use of CCTA is associated with an apparent reduction in fatal and nonfatal MI. Because the overall event rates were low and the absolute number of events small, this has raised 2 specific questions: is this a real effect, and if so, what is the mechanism? These new data help us to address these questions. First, although there was no statistically significant interaction between time and the trial intervention, the event curves did appear to overlap before diverging after approximately 2 months. From first principles, this is not surprising given that our current data clearly demonstrate a time delay from clinic consultation to CCTA conduct and implementation of new therapy. Second, an effect on outcome is not plausible unless the CCTA has led to changes in therapy. We therefore assessed event rates after the median time point where preventive therapies had been prescribed and issued to the patients (50 days) (Central Illustration). This was a conservative estimate given that one-half of patients will still have not received changes in treatment and coronary revascularization procedures took considerably longer to deliver. Despite this, we report a point estimate suggesting a halving in the event rates for fatal and nonfatal MI. Finally, we believe this reduction in events is consistent with the anticipated effect size attributable to initiation of preventive therapies 18, 19, 22, 23, 24 together with potential benefits from life-style modification and coronary revascularization 25, 26. Moreover, it is likely that the early reductions in events will be attributable to antiplatelet therapy 22, 23, with later benefits potentially seen with statin therapy 18, 24 and coronary revascularization procedures 25, 26. Finally, the observed halving of coronary heart disease events is also consistent with our previous findings of improved outcomes following the introduction of better diagnostic pathways in the setting of patients presenting with suspected acute coronary syndrome (27).

**Central Illustration:**
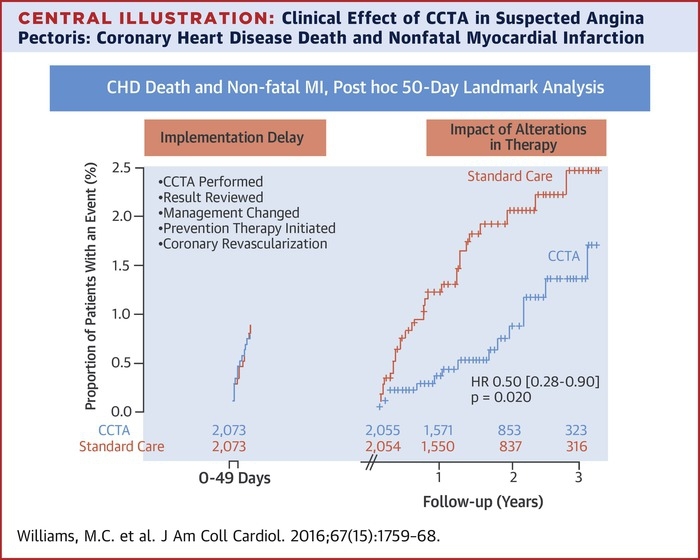
Clinical Effect of CCTA in Suspected Angina Pectoris: Coronary Heart Disease Death and Nonfatal Myocardial Infarction Post hoc landmark analysis at 50 days to account for the implementation and treatment delay consequent on the conduct, reporting, and communication of the coronary computed tomography angiography (CCTA) findings. HR = hazard ratio.

### Study limitations

The major limitation of the present study is the modest number of events, albeit in over 4,000 patients. This reflects the fact that the majority of subjects had no or minimal coronary artery disease and therefore were not at risk. The coronary heart disease (fatal and nonfatal MI) event rate in all patients receiving standard care alone was 2.2% over 20 months. However, the actual rate in those who had significant coronary artery disease was much higher (Online Figure 1) and is consistent with event rates of patients with proven coronary heart disease 18, 19. We intend to continue follow-up for a median of 5 years to allow the accrual of more events that will enable more precise estimates of benefit and facilitate the further exploration of our secondary endpoints.

## Conclusions

We have demonstrated that CCTA facilitates the more appropriate and effective selection of invasive coronary angiography for patients with suspected angina due to coronary heart disease. CCTA also changed the downstream prescribing of preventive therapies and the application of coronary revascularization procedures that were associated with an apparent halving in the rates of fatal and nonfatal MI. Arguably, this is the first time that a noninvasive diagnostic test for coronary heart disease has demonstrated a benefit in hard clinical outcomes through better targeted investigations and treatments in patients presenting with suspected angina pectoris due to coronary heart disease.

Perspectives **COMPETENCY IN PATIENT CARE AND PROCEDURAL SKILLS:** In patients with symptomatic coronary disease, the results of CCTA can guide referral for invasive angiography and revascularization and reduce the risk of MI.

**TRANSLATIONAL OUTLOOK:** Further studies are needed to determine whether guiding clinical management of patients with ischemic heart disease by initial coronary CCTA improves long-term outcomes.
