# Supplementary material for: Use of Coronary Computed Tomographic Angiography to Guide Management of Patients With Coronary Disease
Source: J Am Coll Cardiol. 2016 Apr 19;67(15):1759–68. doi: 10.1016/j.jacc.2016.02.026 (PMC4829708; doi:10.1016/j.jacc.2016.02.026)
Supplement: Online Data [file mmc1.docx]

**SUPPLEMENTARY MATERIAL**

**Impact of Coronary Computed Tomography Angiography on**

**Clinical Management and Outcomes**

**in Patients with Suspected Angina due to Coronary Heart Disease**

**Economic analysis**

Our cost effectiveness analysis was designed to assess the impact of coronary computed tomography angiography (CCTA) on resource use. Resource consequences were measured over a 6-month follow-up period from the date of randomization. All hospital based resource use was included along with selected drugs used to prevent or treat cardiovascular disease. A United Kingdom National Health Service (NHS) perspective was used for measuring and valuing resource costs and an individual perspective was adopted for measuring quality-of-life. Costs were calculated in 2014 GBP (£) and converted to USD ($) using a purchasing power parity of 0.699 ($/£). Analysis of incremental costs was by intention-to-treat with patients grouped according to their randomized allocation to CCTA plus standard care or standard care alone.

***Direct Costs of CCTA***

In our base case analysis, we estimated the distribution of direct CCTA costs using a normal distribution with a mean of £302±50, bounded by values between £210 and £390. Our estimate of £302 follows the report by Genders et al^1^ but revalues their cost estimate to 2014 price levels. We also allowed for alternative estimates of the direct cost of CTA using truncated normal distributions with means of £125 and £550 to encompass uncertainty and the variation in reported CCTA costs between European and North American hospital settings. The lower value is compatible with the micro-costing exercise of CCTA conducted by Darlington et al^2^ and used by Goodacre et al^3^ in their development of an economic model based on NHS reference costs. North American cost estimates tend to be higher, reflecting the early use of 64-slice CT scanners.^4^ The higher mean cost also enables consideration of a pattern of less efficient use of CCTA when throughput is below capacity.

***Hospital Out-patient, Day-case and Inpatient Costs***

The cost of the initial chest pain clinic visit is excluded from the analysis as this component of resource use occurred at patient recruitment before the randomization date. All outpatient attendances (out-patient clinics or non-invasive investigations), day-case services (admission to hospital without an overnight stay) and inpatient episodes (admission to hospital with overnight stay) were recorded not only those associated with further investigation and management of coronary heart disease. Hospital service use was valued using hospital/center and specialty specific costs per attendance, day or duration of hospital stay based on published cost information for NHS Scotland, Scottish Health Service Costs. To allow for variation, we used a discrete distribution to simulate center-specific cardiology outpatient and day-case costs reflecting the empirical distribution of hospital locations where randomized patients received treatment. The cost of all inpatient episodes were calculated by applying Scottish national average specialty-specific per diem costs to the duration of inpatient stay in days. Resource use was truncated at 6 months for the analysis.

***Medication Costs***

The Scottish national drug-prescribing database was used to obtain information on all preventative medications, categorized into anti-platelet, statin and angiotensin-converting enzyme inhibition therapies. Unit costs for drug therapy (derived from the British National Formulary) reflected National Institute for Health and Care Excellence, Scottish Medicine Consortium and Lothian Joint Formulary guidance for first line treatment options.

**Supplementary Results**

**Table S1**

Alterations in medical therapy according to the result of the coronary computed tomography coronary angiography (CCTA).

| **Therapy** | **CCTA results** | | | | | |
| --- | --- | --- | --- | --- | --- | --- |
|  | **Normal** | | **Non-obstructive** | | **Obstructive** | |
|  | **Number** | **%** | **Number** | **%** | **Number** | **%** |
| **Antiplatelet started** | 646 | 99.54 | 530 | 77.94 | 280 | 83.58 |
| **No** |  |  |  |  |  |  |
| **Yes** | 3 | 0.46 | 150 | 22.06 | 55 | 16.42 |
| **Antiplatelet cancelled** | 581 | 89.52 | 678 | 99.71 | 333 | 99.40 |
| **No** |  |  |  |  |  |  |
| **Yes** | 68 | 10.48 | 2 | 0.29 | 2 | 0.60 |
| **Statin started** | 647 | 99.69 | 515 | 75.74 | 284 | 84.78 |
| **No** |  |  |  |  |  |  |
| **Yes** | 2 | 0.31 | 165 | 24.26 | 51 | 15.22 |
| **Statin cancelled** | 621 | 95.69 | 676 | 99.41 | 333 | 99.40 |
| **No** |  |  |  |  |  |  |
| **Yes** | 28 | 4.31 | 4 | 0.59 | 2 | 0.60 |
| **ACE inhibitor started** | 649 | 100.0 | 670 | 98.53 | 329 | 98.21 |
| **No** |  |  |  |  |  |  |
| **Yes** | 0 | 0 | 10 | 1.47 | 6 | 1.79 |
| **ACE inhibitor cancelled** | 648 | 99.85 | 679 | 99.85 | 335 | 100.0 |
| **No** |  |  |  |  |  |  |
| **Yes** | 1 | 0.15 | 1 | 0.15 | 0 | 0 |
| **Beta-blocker started** | 648 | 99.85 | 667 | 98.09 | 311 | 92.84 |
| **No** |  |  |  |  |  |  |
| **Yes** | 1 | 0.15 | 13 | 1.91 | 24 | 7.16 |
| **Beta-blocker cancelled** | 611 | 94.14 | 673 | 98.97 | 334 | 99.70 |
| **No** |  |  |  |  |  |  |
| **Yes** | 38 | 5.86 | 7 | 1.03 | 1 | 0.30 |
| **Antianginal started** | 649 | 100.0 | 662 | 97.35 | 310 | 92.54 |
| **No** |  |  |  |  |  |  |
| **Yes** | 0 | 0 | 18 | 2.65 | 25 | 7.46 |
| **Antianginal cancelled** | 588 | 90.60 | 663 | 97.50 | 334 | 99.70 |
| **No** |  |  |  |  |  |  |
| **Yes** | 61 | 9.40 | 17 | 2.50 | 1 | 0.30 |
| **Preventative started** | 645 | 99.38 | 473 | 69.56 | 264 | 78.81 |
| **No** |  |  |  |  |  |  |
| **Yes** | 4 | 0.62 | 207 | 30.44 | 71 | 21.19 |
| **Preventative cancelled** | 575 | 88.60 | 670 | 98.53 | 332 | 99.10 |
| **No** |  |  |  |  |  |  |
| **Yes** | 74 | 11.40 | 10 | 1.47 | 3 | 0.90 |

**Table S2**

Prevalence of revascularization and preventative therapies in patients who had fatal or non-fatal myocardial infarction after the 50-day landmark.

|  | **Total** | **Revascularization**  **Prior to Event** | | | **Preventative Therapy**  **Prior to Event** | | |
| --- | --- | --- | --- | --- | --- | --- | --- |
|  |  | **Yes** | **No** | **% Without** | **Yes** | **No** | **% Without** |
|  |  |  |  |  |  |  |  |
| **Coronary Computed Tomography Angiography + Standard Care** | 17 | 13 (11 + 2)* | 4 | 24% | 15 | 2 | 12% |
| **Standard Care Alone** | 34 | 21 (18 + 3)* | 13 | 38% | 27 | 7 | 21% |
|  |  |  |  |  |  |  |  |

*(Percutaneous Coronary Intervention + Coronary Artery Bypass Graft Surgery)

**Figure S1**

Three-year rates of coronary heart disease events in patients who underwent coronary computed tomography angiography (n=1,778) according to the presence of coronary artery disease.

**
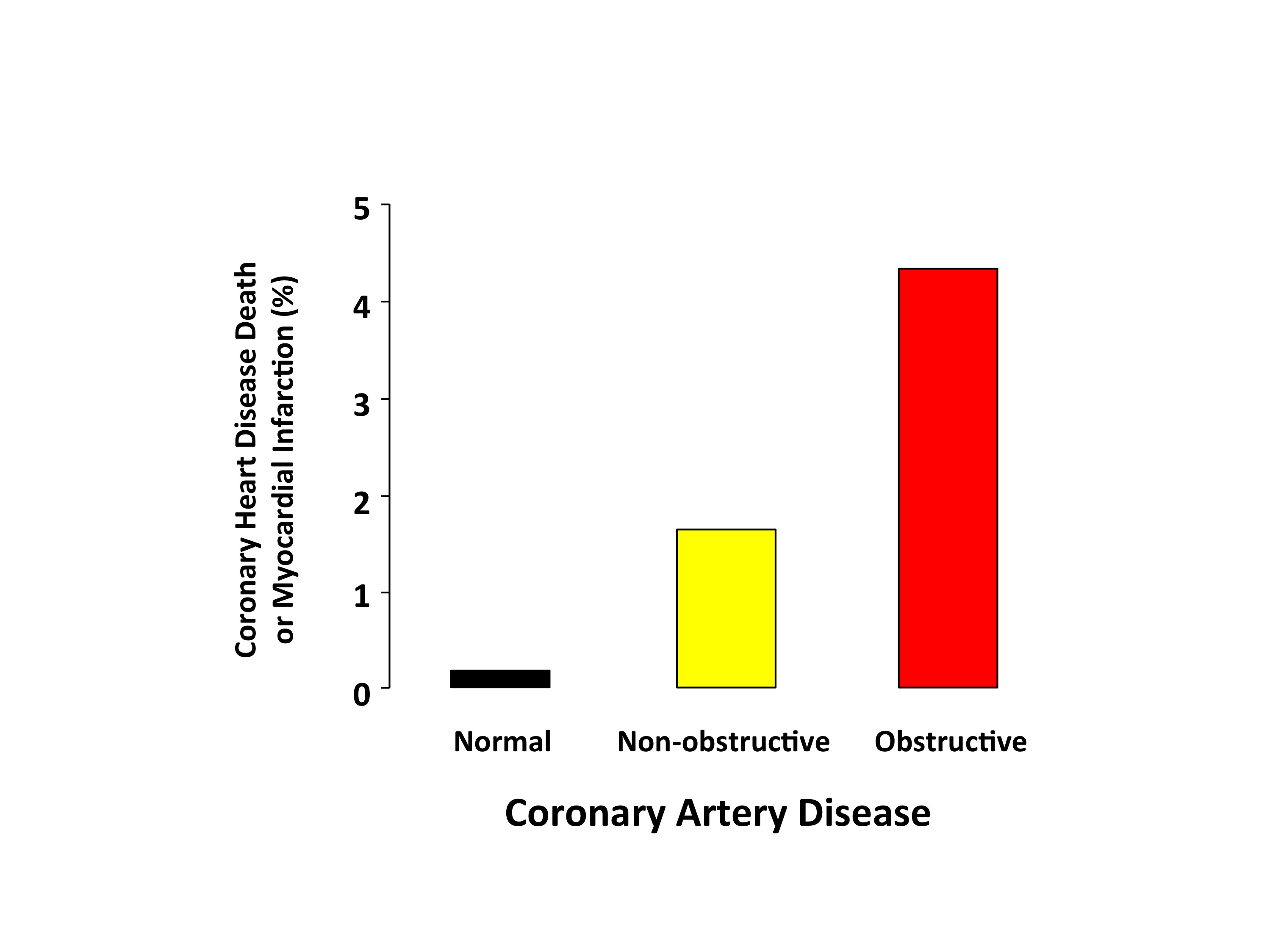
**

**References**

1. Genders TS, Petersen SE, Pugliese F, Dastidar AG, Fleischmann KE, Nieman K, Hunink MG. The optimal imaging strategy for patients with stable chest pain: a cost-effectiveness analysis. *Ann Intern Med.* 2015;162:474-84.

2. Darlington M, Gueret P, Laissy JP, Pierucci AF, Maoulida H, Quelen C, Niarra R, Chatellier G, Durand-Zaleski I. Cost-effectiveness of computed tomography coronary angiography versus conventional invasive coronary angiography. *Eur J Health Econ.* 2015;16:647-55.

3. Goodacre S, Thokala P, Carroll C, Stevens JW, Leaviss J, Al Khalaf M, Collinson P, Morris F, Evans P, Wang J. Systematic review, meta-analysis and economic modelling of diagnostic strategies for suspected acute coronary syndrome. *Health Technol Assess.* 2013;17:v-vi, 1-188.

4. Ladapo JA, Jaffer FA, Hoffmann U, Thomson CC, Bamberg F, Dec W, Cutler DM,Weinstein MC, Gazelle GS. Clinical outcomes and cost-effectiveness of coronary computed tomography angiography in the evaluation of patients with chest pain. *J Am Coll Cardiol.* 2009;54:2409-22.
